# Design advanced lithium metal anode materials in high energy density lithium batteries

**DOI:** 10.1016/j.heliyon.2024.e27181

**Published:** 2024-02-29

**Authors:** Ran Tian, Jingyu Jia, Meixiang Zhai, Ying Wei, Xinru Feng, Ruoqi Li, Jinyan Zhang, Yun Gao

**Affiliations:** a Fujian Nanping Nanfu Battery co., ltd, Nanping, Fujian, 353000, China; b Department of Materials Science and Engineering, University of Science and Technology of China, Hefei, Anhui, 230026, China; c College of Chemical Engineering, North China University of Science and Technology, Hebei, 063210, China; d State Key Laboratory of Advanced Brazing Filler Metals and Technology, Zhengzhou Research Institute of Mechanical Engineering Co., Ltd. Zhengzhou,450001, China

**Keywords:** Lithium battery, Lithium metal anode, Nano-interface engineering

## Abstract

Nowadays, the ongoing electrical vehicles and energy storage devices give a great demand of high-energy-density lithium battery. The commercial graphite anode has been reached the limit of the theoretical capacity. Herein, we introduce lithium metal anode to demonstrate the promising anode which can replace graphite. Lithium metal has a high theoretical capacity and the lowest electrochemical potential. Hence, using lithium metal as the anode material of lithium batteries can reach the limit of energy and power density of lithium batteries. However, lithium metal has huge flaw such as unstable SEI layer, volume change and dendrites formation. Therefore, we give a review of the lithium metal anode on its issues and introduce the existing research to overcome these. Besides, we give the perspective that the engineering problems also restrict the commercial use of lithium metal. This review provides the reasonable method to enhance the lithium metal performance and give the development direction for the subsequent research.

## Graphical abstract

**Figure:**
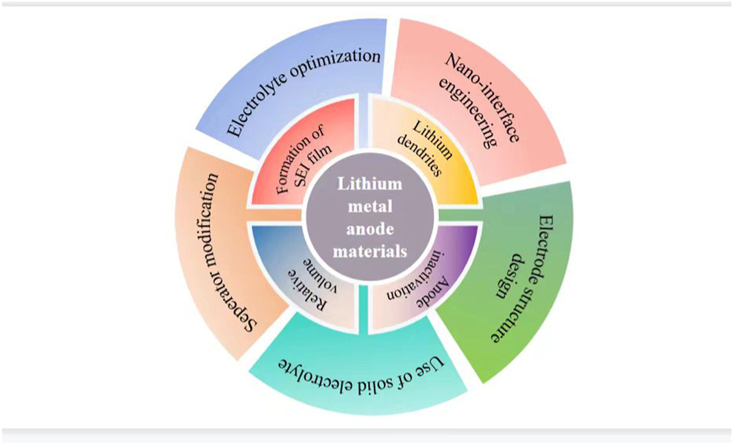

## 1 Principle of lithium battery

**Fig. 1:**
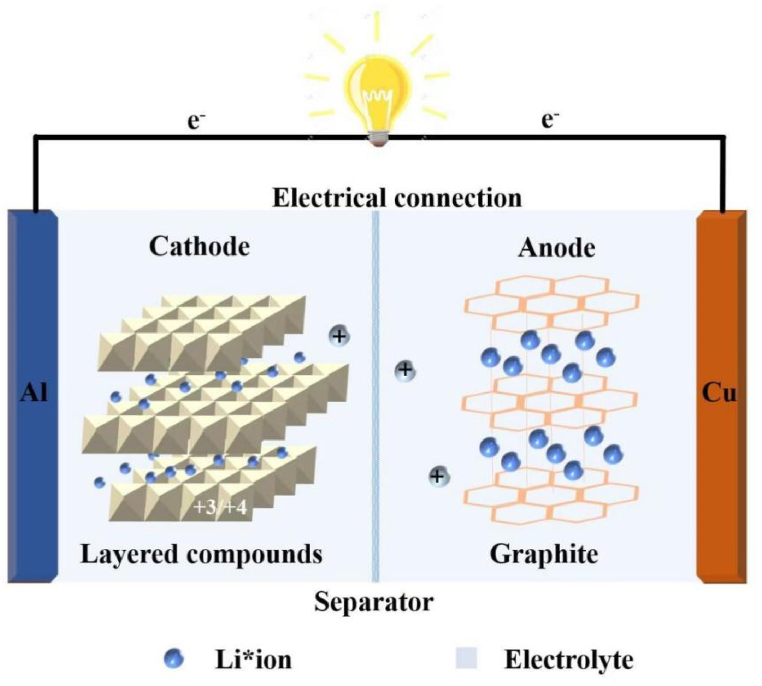
Electrochemical mechanism of commercial lithium battery.

Lithium battery refers to electrochemical energy storage batteries with Lithium elements (including Lithium metal, Lithium alloy, Lithium ion and Lithium polymer) [[1], [2], [3], [4], [5], [6], [7], [8], [9], [10]]. As an electrochemical energy storage device, lithium battery can convert chemical energy and electric energy into each other through electrochemical reaction [[11], [12], [13], [14], [15], [16], [17]]. The energy storage mechanism of lithium battery is very simple. It makes use of electric energy to intercalate (de-intercalate) lithium ions on the electrode, so that reduction (oxidation) reaction takes place [[18], [19], [20], [21], [22], [23], [24], [25]]. A basic structure of lithium battery is shown in Fig. 1, which uses graphite (C_6_) and lithium cobalt oxides (LiCoO_2_) as the anode and cathode materials of the battery respectively to show the electrochemical reactions occurring in the charging and discharging process of commercial lithium battery [26,27]. The specific reaction equation is as follows.

Cathode: (Equations 1-1)

Anode: (Equations 1-2)

The whole reaction: (Equations 1-3)

In the charging process, the layered LiCoO_2_ is oxidized, the 3-valent cobalt ion is converted to 4-valent cobalt ion, and lithium ion is de-intercalated from the cathode to form Li_1-x_CoO_2_(Equation 1-1) [28,29], while the electrode is reduced in this process. Electrons enter the antibonding orbital of the graphite layer to form C_6_^−^, and lithium ion is intercalated between the lamellar graphite [30]. The ionic compound Li_x_C_6_(Equation 1-2) is formed. In the process, electrical energy is converted into chemical energy.

The reaction in the discharge process is the reverse reaction in the charging process. At this time, Li_1-x_CoO_2_ is intercalated into lithium ions to form LiCoO_2_, while the lithium ions in Li_x_C_6_ are de-intercalated to form graphite. In this process, chemical energy is converted to electric energy [31,32]. The total reaction formula of commercial lithium battery can be expressed by Equations 1-3.

Lithium ion batteries made of graphite and lithium cobalt oxides are known as rocker batteries because of their electrochemical reversibility, allowing lithium ions to migrate between the anode and cathode materials with little loss [[33], [34], [35], [36], [37], [38], [39], [40]]. Since the potential gap between anode and cathode reaches 3.7 V, the energy density of commercial lithium ion batteries has obvious advantages over low-voltage batteries such as nickel metal hydride and nickel-cadmium batteries, and almost reaches the electrochemical limit [[41], [42], [43]]. The energy density of the lithium battery can reach 140 Wh kg^−1^ and 200 Wh L^−1^ in the graphite-lithium cobalt oxides system. However, the ongoing electrical vehicles and energy storage devices give a great demand of high energy density lithium battery which can promote the development the next generation of anode materials [[44], [45], [46]]. In this review, we mainly introduce the reactive anode materials and lithium metal which have high specific capacity and low electrochemical potential and provide our opinions to design the anode materials in high energy density lithium battery.

## 2 Lithium metal anode introduction

Lithium metal is one of the candidate anode materials for the next generation of lithium batteries [[47], [48], [49], [50], [51], [52], [53], [54], [55], [56]]. As an alternative to the traditional carbon anode, lithium metal has a theoretical capacity of 3860 mAh g^−1^, the lowest electrochemical potential (−3.04 V vs standard hydrogen electrode). Therefore, using lithium metal as the anode material of lithium batteries can reach the limit of energy and power density of lithium batteries [54,[57], [58], [59]].

The use of lithium metal as the anode material of lithium secondary batteries began in the early 1970s [60]. Whittingham et al. first invented lithium secondary batteries using lithium metal as the anode. However, until now, lithium metal anode has not been used commercially on a large scale, mainly due to its safety and cycle stability [[61], [62], [63]]. During this time, the carbon material anode was invented and rapidly replaced the lithium metal anode [64,65]. This kind of lithium free anode and lithium-rich cathode such as lithium cobalt oxides (LiCoO_2_) and lithium iron phosphate (LiFePO_4_) form an excellent match [65,66]. Solid electrolyte film formed by carbon anode material can inhibit the occurrence of electrode side reactions and provide a voltage window of 4V or larger [67]. However, the main reason that restricts the development of lithium batteries is the lack of energy density.

**Fig. 2:**
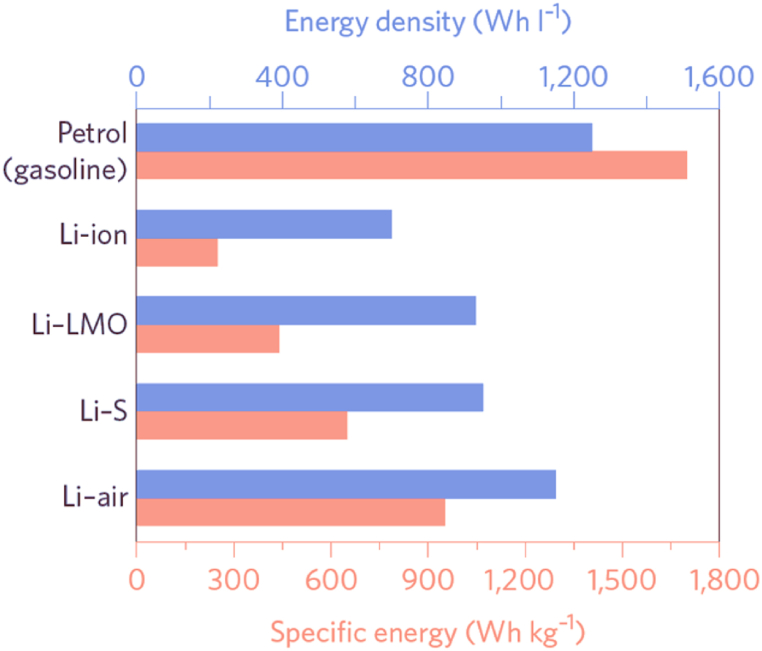
Energy densities for different systems LIBs [72].

At this stage, to use commercial lithium-ion batteries due to its cathode materials and the cathode material of lithium storage ability is bad, in terms of energy density is far lower than the theoretical energy density of lithium metal batteries (Fig. 2), so the new systems with lithium metal anode, such as lithium sulfur batteries [68,69], lithium air batteries [70,71] due to their high energy density theory, have attracted the attention of the researchers. By using lithium metal battery, the electric cars can have longer travlled distance and cellphones have more service time which can promote the scientific and technical revolution.

## 3 Challenges of lithium metal anodes

### 3.1 Formation of solid electrolyte interface (SEI) film

Since the potential of Li^+^/Li is low, almost all electrolytes can be reduced by lithium metal to form a SEI film on the surface of lithium metal, so the study of SEI film is an important part of the study of lithium metal in liquid electrolyte [[73], [74], [75], [76], [77], [78], [79]]. Due to the passivation effect of SEI film on the surface of lithium metal, lithium metal battery can work stably and provide a voltage window over 4V [[80], [81], [82]]. The need in the aspect of composition, structure and ionic conduction properties have good uniformity [83,84]. Because the loop around the surface morphology of metallic lithium tremendous changes will occur, the SEI film also needs to have good flexibility, even need a degree of elasticity [85,86].

Carbonate electrolyte is the most widely used electrolyte type for commercial lithium batteries, but it is not the ideal electrolyte for lithium metal anode [87]. This is because the initial SEI film on the surface of lithium metal is mainly composed of lithium alkyl carbonate (ROCOOLi), which is generated by one-electron reduction of electrolyte solvent. In the presence of trace amounts of water, alkyl lithium carbonate will be further converted to lithium carbonate (Li_2_CO_3_) [88]. Since lithium metal also generates byproducts of other inorganic components such as LiF and Li_2_O during the reaction process, these inert inorganic substances often exist in the interior of SEI film, while metastable ROCOOLi usually exists in the exterior of SEI film [89]. Therefore, this SEI film can be regarded as a mosaic structure formed by a variety of components and has obvious phase separation, so it does not have good flexibility. In the process of charging and discharging, it is easy to form stripping, leading to the increase of impedance of lithium metal anode and inactivation [90].

Ether electrolyte is a kind of lithium metal anode-friendly electrolyte which can effectively improve the Coulombic efficiency of lithium metal anode and inhibit the growth of lithium dendrites [91]. This is mainly because the oligomer SEI film generated by the reaction with lithium metal has good flexibility, but the low electrochemical window (less than 4 V) and high flammability of ether electrolyte restrict its application in commercial lithium batteries.

### 3.2 Formation of lithium dendrites

Dendrite formation is a common industrial phenomenon in electrochemically deposited metals, and the mechanism of dendrite growth has been thoroughly studied. In electrochemical deposition process, the cation in the electrolyte between the two electrodes will exist in a certain concentration gradient, once the current reaches the critical current density [50,92], the current will only be able to maintain a certain amount of time, this time called Sand's time τ, cationic will reduce gradually, after breaking the balance of deposition on the surface and the electrode electric field. It leads to the formation of space charge layer and changes the morphology of metal electrodeposition [93,94]. This theory well predicts that lithium metal will grow as dendrite when the current density is greater than limited current J. However, in the actual test, when the deposition current is less than J, the formation of dendrites will also be observed. Therefore, this mechanism cannot fully explain the cause of dendrite formation, mainly because it does not take into account the factors of interface chemistry.

Similar to other highly active metals, lithium metal also spontaneously generates SEI film on the surface. In the Mosaic model, this SEI layer shows inhomogeneous lithium ion conduction and leads to inhomogeneous nucleation of lithium metal. In addition, the huge volume change of lithium metal in the cycling process will lead to the rupture of SEI layer [95,96]. The newly exposed lithium metal has a low resistance to lithium ion transport, and the improvement of ion conduction capacity will aggravate the uneven deposition of lithium metal. Therefore, the dendrite growth of lithium metal is self-reinforcing, and the curvature of the electric field line in the protruding part will increase, which will attract more lithium ions, thus leading to the preferential deposition of lithium metal in the protruding part and the formation of lithium dendrites.

### 3.3 Relative volume change

All electrode materials of lithium ion batteries will have a certain volume change during the insertion/removal of lithium. For commercial graphite anode materials, there will also be a volume change of about 10% during the cycle. For alloy anode materials, such as silicon, there will be a volume change of about 400% before and after the cycle, which greatly affects their use [97,98]. For lithium metal, since there is no substance for lithium storage, it can be considered that the volume change of lithium metal is infinite. From the perspective of practical use, the single side commercial electrode needs to have a capacity of at least 3 mAh cm^−2^, which is equivalent to the thickness change of metallic lithium of 14.6 μm [99]. If the battery capacity becomes larger, the volume change of lithium metal anode can reach tens of microns, which will greatly test the stability of SEI film and affect the battery safety.

### 3.4 Causes of lithium metal anode inactivation

At present, the most important problems faced by lithium metal electrodes are the instability of the interface between lithium metal and electrolyte and the dendrite growth on the anode surface, which will lead to safety hazards, low utilization rate, short service life and other problems of lithium metal anode.

**Fig. 3:**
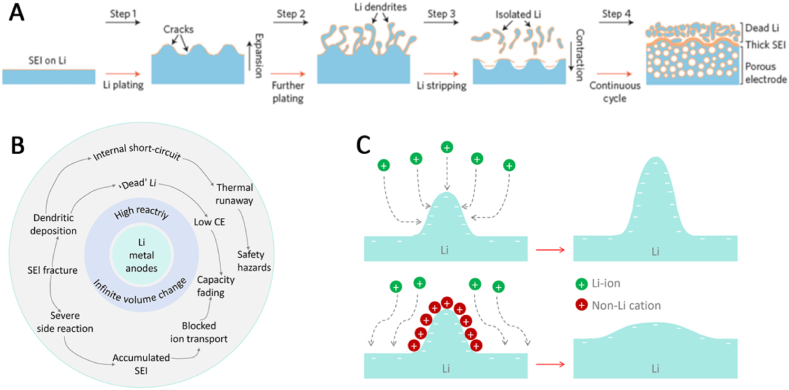
Schematic showing the Li stripping/plating process (A), Correlations among the different challenges in the Li metal anode, originating from high reactivity and infinite relative volume change (B), Schematic illustration of the Li deposition process based on the self-healing electrostatic shield mechanism(C) [72].

In actual use, the cause for silencing of lithium metal anode can be summarized as shown in Fig. 3(A). Because of the high reactivity of metallic lithium and infinite volume change, the electrochemical reaction will cause the SEI layer on the lithium metal break, resulting in the formation of lithium dendrites, the increasing of the electrochemical reaction, and the production of lithium. The cycle lead to lithium metal gradual deactivation, and can come with serious safety issues and battery capacity decay [100,101]. The specific deactivation process is shown in Fig. 3 B. During the deposition of lithium metal, huge volume changes will occur, which will lead to the rupture of SEI layer on the surface of lithium metal, and lithium dendrites will preferentially grow at the fracture site [100]. In the stripping stage of lithium metal, the root of dendrite is prone to fracture, which makes it out of electrical contact with the conductor, and then forms dead lithium. In the subsequent cycle, this process occurs continuously, which will lead to the formation of porous lithium on the surface of lithium metal [102]. This process will be accompanied by a large number of broken SEI layer and dead lithium, which will block the transmission of ions on the electrode surface, thus leading to the attenuation of capacity. Therefore, the development and use of lithium metal anode is still a great challenge at the present stage [103].

In summary, effective regulation of lithium ion deposition behavior and effective inhibition of dendrite growth are the most urgent problems to be solved in the practical application of lithium metal batteries [104]. These are common challenges for lithium metal batteries, each of which is difficult to deal with.

## 4 Improvement of lithium metal anode

### 4.1 Liquid electrolyte optimization

The addition of electrolyte additives is one of the effective methods to improve the performance of lithium metal anode. These additives can change the physical and chemical properties of SEI layer on lithium metal surface by decomposition, polymerization or adsorption, so as to adjust the local current density in the process of lithium metal deposition. Some electrolyte additives, even at PPM level, can change the deposition morphology and improve the stability of the cycle [105]. Classical electrolyte additives include 2-methylfuran [106], organic aromatic compounds [107], ethylene carbonate [108], and some surfactants [109]. However, this method can only restrain the lithium dendrites growth and prolong the cyclic life. It can't solve the problem in basics.

**Fig. 4:**
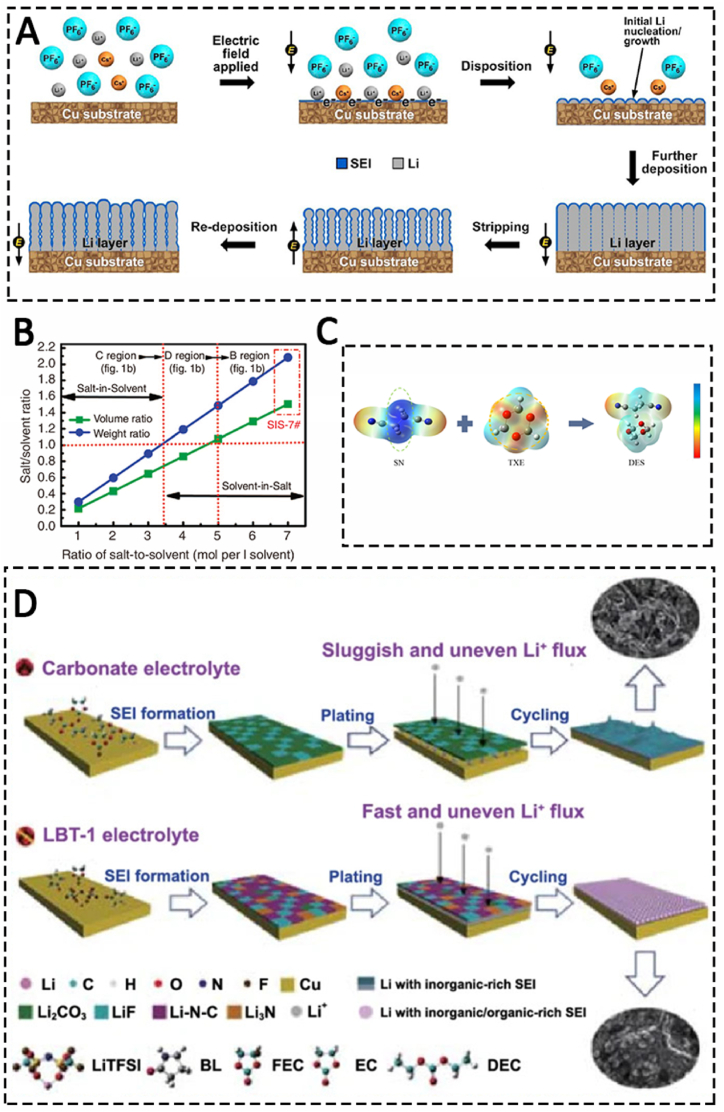
Schematic of Li deposition and stripping processes when the Cs additive is used (A) [111]. Weight and volume ratio of salt-to-solvent with different ratios of LiTFSI to DOL:DME (1:1 by volume) (B) [112]. Schematic of the deep eutectic electrolyte forming (C) [113]. The SEI layer structure in DEE (D) [114].

The optimization principles are not influencing the ionic conductivity and electrochemical window. The other rule is that the new electrolyte can effectively change the lithium ion movement on the anode. Electrostatic shielding electrolyte additives are one type of the additives for lithium metal anode electrolyte, which are commonly soluble salts of Cs^+^ and Rb^+^ [110,111]. These alkali metal ions can inhibit the growth of lithium dendrites by virtue of their self-healing electrostatic effects (Fig. 3(C)). According to Nernst equation, if the reduction potential of metal ion (M^+^) is close to that of lithium ion, and its concentration is much lower than that of lithium ion, then M^+^ has a lower effective reduction potential than lithium ion. Therefore, in the process of lithium metal deposition, M^+^ ions are adsorbed on the surface of lithium metal without being reduced. If lithium metal is deposited unevenly, the charge will accumulate in the protrusion of the deposit, which will attract more M^+^ ions and create an electrostatic shielding electric field there (Fig. 4(A)) [111]. The positive charge shielding can hinder the transfer of lithium ions, thus reducing the deposition rate of lithium metal in the protrusion, inhibiting the growth of lithium dendrite in the deposition process, and realizing the uniform deposition of lithium metal.

High concentration lithium electrolyte is also one of the effective methods of electrolyte optimization [115,116]. In the classical model of ideal lithium metal deposition, high concentration of lithium electrolyte can increase the current threshold J of lithium metal dendrite deposition, thus inhibiting the formation of lithium dendrites. Hu and co-researchers invented a lithium bistrifluoromethylsulfonimide (LiTFSI) electrolyte with electrolyte concentration of 7 M, and it was successfully applied in the lithium-sulfur battery system [112]. The electrolyte with high concentration of lithium ion can effectively inhibit the production of lithium dendrites, the dissolution of polysulfide, and improve the cycle performance of Li–S batteries. More importantly, this electrolyte greatly improves the migration number of lithium ions, mainly because the high concentration of lithium salt will bring non-solvated lithium ions, so it also improves the high-rate performance of the battery (Fig. 4(B)). Similarly, Zhang et al. realized tumorous lithium metal deposition in ether electrolyte with 4 M lithium difluoride sulfonimide (LiFSI), and achieved high coulombic efficiency at high rate. Although high concentration lithium ion electrolyte can make lithium metal have safer and more stable performance, the cost increase brought by it also needs further improvement [117].

Recently，eutectic solution gives the highly stable cyclic performance of lithium metal anode because it remove the solvation shell of Li^+^ in the electrolyte. Cui found a new type of deep eutectic electrolyte (DEE) with a thermally induced smart shut-down function. It is presented to ameliorate the aforementioned issues (Fig. 4(C)) [113]. In this delicately designed DEE, 1,3,5-trioxane (TXE) can participate in the Li^+^ primary solvation shell and form an unique solvation structure (Li^+^-SN-TXE-DFOB^−^), which is favorable to enhance the Li/electrolyte interfacial compatibility. Zhang et al. introduced an emerging amide-based electrolyte containing LiTFSI and butyrolactam in different molar ratios. 1,1,2,2-Tetrafluoroethyl-2,2,3,3-tetrafluoropropylether and fluoroethylene carbonate into the amide-based electrolyte as counter solvent and additives. An inorganic/organic-rich solid electrolyte interphase with an abundance of LiF, Li_3_N and Li–N–C is in situ formed, leading to spherical lithium deposition without dendrites formation (Fig. 4(D)) [114].

### 4.2 Nano-interface engineering

The stability of SEI film plays an important role in the performance of lithium metal anode, and the thickness of SEI film is only nanometer level, so the research and improvement of the nano-interface of SEI film is the main challenge of lithium metal anode. The ideal structure of SEI film should have the characteristics of nanometer thickness, uniformity and stability, high ionic conductivity and superior mechanical properties.

**Fig. 5:**
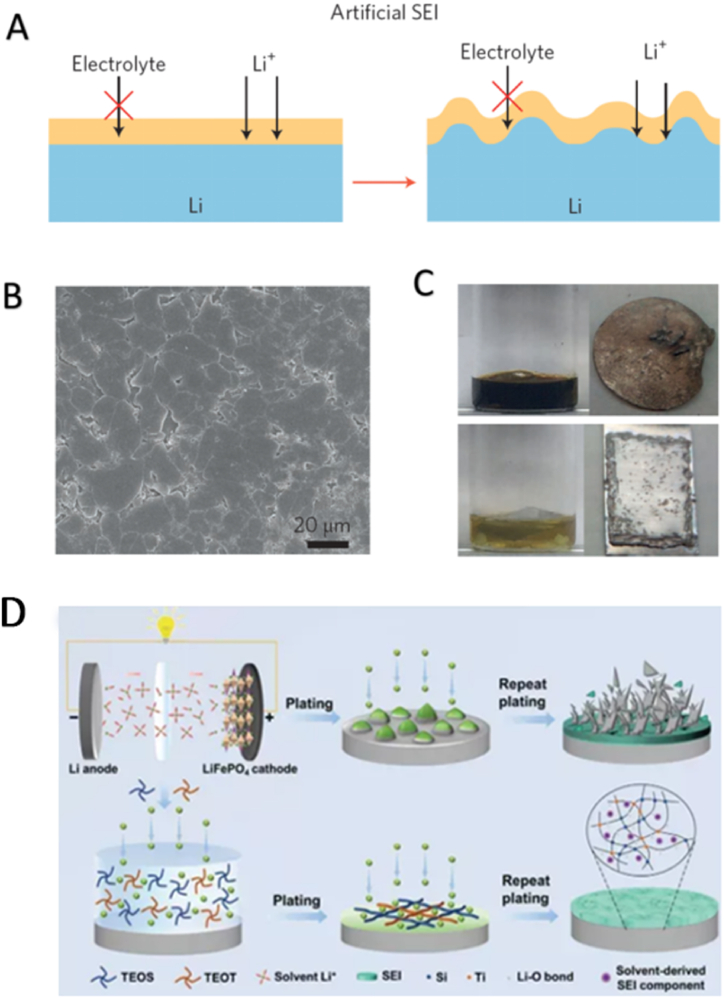
Schematic showing the design principles and mechanisms of artificial SEI(A) [80]. Top-view SEM images of Li_3_PO_4_-modified Li anode(B) [122]. Optical images of pristine (top) and 14 nm ALD Al_2_O_3_-protected (bottom) Li metal foil(C) [123]. Interfacial properties of Li anodes with TEOS and TEOT electrolyte additives(D) [124].

An effective way to improve the anode performance of lithium metal is to construct an artificial SEI film on the surface of lithium metal, which can form a physical barrier on the surface of lithium metal. After theoretical demonstration, when the strength of this film reaches GPa level, it can effectively hinder the growth of lithium dendrites. Moreover, it has been recently proved that when the interfacial tension and ion transport on lithium metal surface change, the strength of artificial SEI film reaches tens of MPa, which is enough to inhibit the growth of lithium dendrites [113,114]. Such artificial SEI layer are generally chemically inert and compact, thus preventing corrosion of lithium metal by the electrolyte, and have suitable lithium ion conduction capabilities (Fig. 5(A)) [110]. There are many ways to form artificial SEI films. Firstly, lithium metal can be formed by exposing it to a specific atmosphere. For example, organic matter replacing silane or having hydroxyl (-OH) groups can react with lithium metal to form a stable protective layer on the surface of lithium metal [118,119]. Have the protective layer of lithium metal anode in the organic electrolyte has minimal initial resistance, and the impedance of the battery in the process of circulation of relatively slow growth. The use of poly-silane as precursor of lithium silicate artificial SEI method can very effectively improve the service life of the lithium battery [120]. In addition, adding ionic liquid or other reactive additives to the electrolyte can also form SEI layer with special structures on the surface of lithium metal [121]. Guo reacted lithium metal in dimethyl sulfoxide solution of poly-phosphate and formed a 50 nm dense Li_3_PO_4_ film on the surface of lithium metal. As this dense SEI layer has extremely high Young's modulus and good lithium ion conductivity, after 200 cycles of charging and discharging, the surface of lithium metal still has a flat morphology, and there is no obvious dendrite (Fig. 5(B)). However, the generation of an artificial SEI film on the surface of lithium metal by direct reaction often requires very precise control of reaction conditions and reactants, so that the generated artificial SEI layer can have good electrochemical performance [122].

In addition, advanced film preparation technology is also considered to be able to effectively change the state of lithium metal surface, inhibit the corrosion of lithium metal by air and active components in the electrolyte. However, since the melting point of lithium metal is very low, it is very difficult to realize film deposition on lithium metal [125]. Moreover, it is necessary to realize the uniform properties of the film, less defects and the control of thickness. For example, magnetron sputtering technology can be used to deposit LPON film on the surface of lithium metal to improve the stability of lithium metal anode [126]. Atomic layer deposition (ALD) technology is also widely used as the optimal choice for the preparation of high quality nano thickness at low temperature [[123], [125], [126], [127]]. The application of ALD technology to deposit nano-thickness metal oxides or metal sulfides with ionic conductivity on the surface of lithium metal has been proved to be effective in prolonging the service life of lithium metal anode. For example, Kozen deposited 14 nm of alumina on the surface of lithium metal at low temperature by atomic layer deposition technology. Lithium metal with alumina deposition can be stable in the air for 20 h, and can well prevent the erosion of lithium metal by electrolyte and polysulfide (Fig. 5(C)) [123]. Zhong et al. make a robust SEI connected by poly (titanium siloxane) (PTS) which was constructed by in-situ condensation reaction of TEOS/TEOT electrolyte additives. By this way, Si–*O*–Si bonds on the lithium metal anodes provide fast lithium ion transportation and enhance the battery life [124]. (Fig. 5(D)）

In addition, directly covering lithium metal with a scaffold-like film is also a good way to improve the performance of lithium metal. This scaffold needs to have nano-structure, chemical stability, high mechanical strength and other characteristics to enhance the performance of SEI layer in the recycling process [128]. SEI film will form on the top of the scaffold material. Theoretically, the two will move together, and SEI film will not break. Lithium ions are free to pass through the scaffolding and can occur under it without producing lithium dendrites. Cui and his co-workers used nano hollow carbon shells to cover the surface of copper fluid-collecting, and lithium metal was deposited between the carbon shell and copper foil in a columnlike morphology, without producing lithium metal dendrites. The use of such scaffolds can effectively improve the coulombic efficiency of lithium metal batteries and increase the cycle life [129]. In addition, 2D hexagonal boron nitride (h-BN) and graphene coated on copper foil can achieve a similar effect, resulting in the improvement of lithium metal anode performance [128].

### 4.3 Electrode structure design

**Fig. 6:**
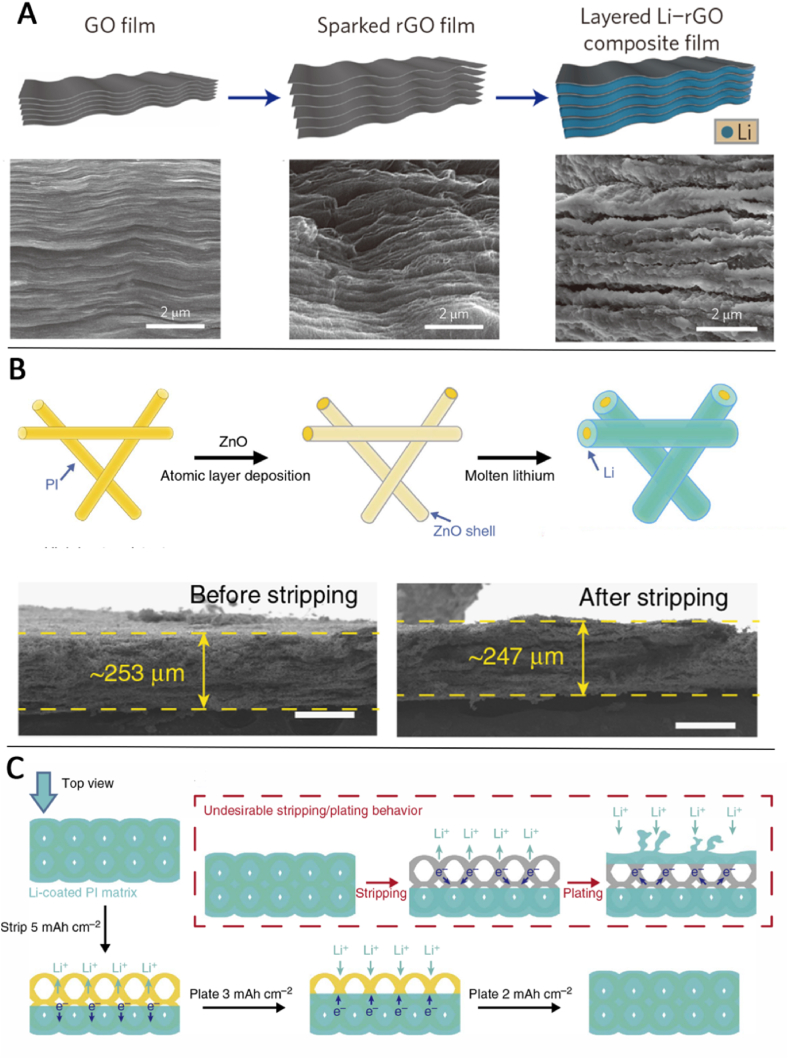
Schematic and corresponding SEM images of the materials layered Li–rGO composite film (right) (A) [132]. Electrospun polyimide (PI) coated with ZnO by ALD to form core–shell PI–ZnO. The ZnO coating produces a ‘lithophilic’ matrix into which molten Li can steadily infuse (B). Schematic illustrating the alternative undesirable Li stripping/plating behaviour where, after stripping, Li nucleate on the top surface, leading to volume change and dendrites shooting out of the Matrix (C) [133].

Although the large volume changes of lithium metal in circulation are one of the important reasons for the decline of electrochemical properties of lithium metal, this problem cannot be completely solved [85,130,131]. Recently, in order to solve this problem, Cui introduced the host material and used the method of pre-intercalated lithium metal to form the lithium composite anode [132]. This lithium metal composite anode can optimize its performance well in lithium air, lithium sulfur and other lithium metal anode systems. For example, by contacting hot melt lithium metal and thin graphene oxide (GO) films, a reduction graphene (rGO) thin films with excellent lithium metal affinity can be prepared (Fig. 6(A)). The edge of the rGO film is in contact with hot melt lithium metal, and the liquid lithium metal will be introduced into the rGO film by capillary, and the composite anode of lithium metal-rGO is prepared. The volume change of this composite anode before and after the cycle is less than 20%, and it can provide stable cyclic performance, low cycle overcurrent and inhibition of lithium dendron growth [132]. This method has a variety of advantages.

(1) Stable host materials can be divided into small parts to better inhibit the volume change before and after lithium metal circulation;
(2) The increase of active lithium metal area can effectively reduce the current density per unit area, and then inhibit the growth of lithium dendrites;
(3) The reduction of volume change can effectively reduce the stress change in the battery during charging and discharging, and reduce the possibility of safety problems in the battery.

The compatibility with lithium metal is a necessary condition for lithium metal composite anode as a carrier. However, unlike GO, most materials and lithium metal are very poor in infiltration, so it is a reasonable improvement method to modify the surface of materials to improve the infiltration of materials and lithium metal. The main surface improvement materials include Si [134], ZnO [133], etc., mainly because these materials can be allogated with lithium metal (lithium silicon alloy and lithium zinc alloy, etc.), so that the coating at the interface can be closely combined with lithium metal. Cui Yi et al. used atomic layer deposition to deposit nanometer thickness ZnO (Fig. 6(B)) on the surface of polyimide fiber, which made the polyimide fiber originally deformable have good affinity with lithium metal. Then the lithium metal melt into this three-dimensional network structure can be obtained, which can significantly reduce the volume change in the cycle and improve the cyclic stability of anode materials (Fig. 6(C)) [133]. These structure design ways need the precision dimension control which has a long way to be large-scale used.

### 4.4 Separator modification

Separator is the key point of the lithium metal battery and provides the electrical isolation between the electrode. In addition, the surface of separator can provide unique property which influence the property of lithium metal anode.

**Fig. 7:**
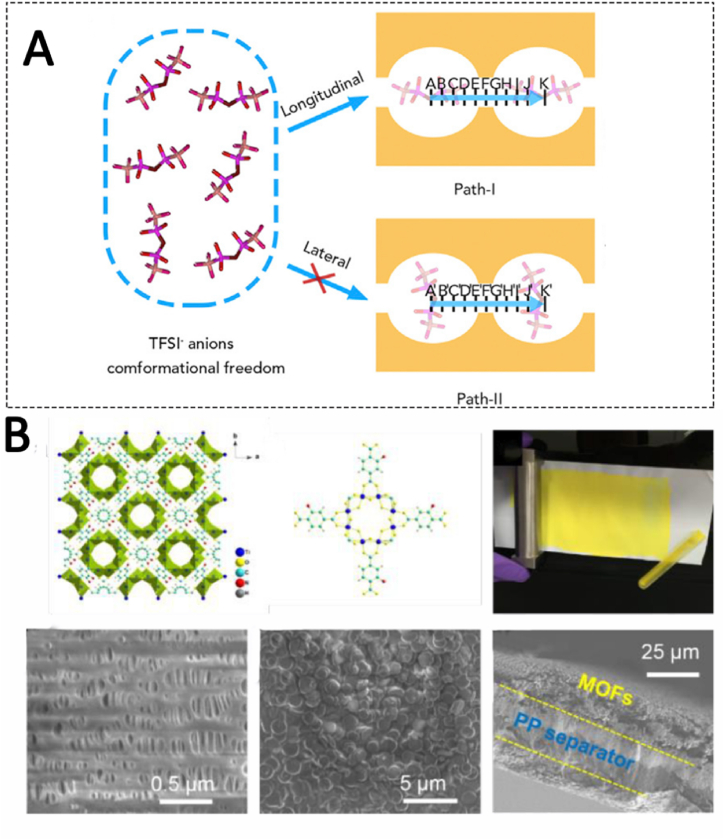
Schematic showing conformational TFSI anion migration between adjacent MOF pores along two different paths (A) [135]. MOF-decorated separator (B) [136].

For example, Zhou mixed HKUST-1 MOF and Graphite oxide to form a composite separator by suction filtration. It was found that the ordered ultra microporous structure of HKUST-1 was used as an ion sieve to achieve effective regulation of anion and cation transport in ordinary electrolyte (1 M LiTFSI DOL/DME 1:1), and showed high ion mobility coefficient and high ion conductivity. Compared with the disordered transport of anions and cations in common electrolyte and uneven lithium deposition, MOF structure can provide an efficient Ion channel, selectively slow down the passage of TFSI^−^ anions in it, so as to achieve uniform lithium ion transport effect and achieve homogeneous metal lithium deposition [135](Fig. 7(A)).Wang et al. used NH_2_-MIL-125 (Ti) to coat the membrane, which increased the lithium ion migration number of the electrolyte from 0.49 to 0.64, and effectively suppressed the formation of lithium dendrites (Fig. 7(B)) [136]. Liu and others made the unique separator with the pre-modification of high-temperature PEN porous membrane in polydopamine (PEN@PDA). Continuous rigid MOF layers are grown in situ on both sides to regulate the distribution of lithium ion flux and suppress the growth of lithium dendrites because the anionic properties and clear inherent microporous structure of the MOF layer on the membrane increase the migration number of lithium ions from 0.22 to 0.81 [137].

### 4.5 Use of solid electrolyte

The development of advanced solid electrolyte is one of the important methods to prevent the side reaction between lithium dendrite and lithium metal, because solid electrolyte can directly form a physical barrier to the growth of lithium dendrites, thus inhibiting the infinite growth of lithium dendrites. Solid electrolytes are mainly divided into two categories: inorganic ceramic-based electrolytes and solid polymer-based electrolytes. Inorganic solid electrolyte is mainly a variety of inorganic lithium ion conductors, such as sulfides [138,139], oxides [140,141], nitrides [142,143]and phosphides [144,145], etc. Polymer solid electrolyte is mainly lithium salt mixed with polymer [[146], [147], [148], [149]]. Solid electrolytes for lithium ions generally need to meet the following criteria.

(1) Sufficient physical strength to prevent lithium dendrites from piercing solid electrolyte.
(2) It has sufficient conductivity of lithium ions at room temperature.
(3) It has a large enough electrochemical window and will not react with anode and cathode materials.
(4) It has a small interface impedance with the electrode, and has a good adhesion with the electrode.

**Fig. 8:**
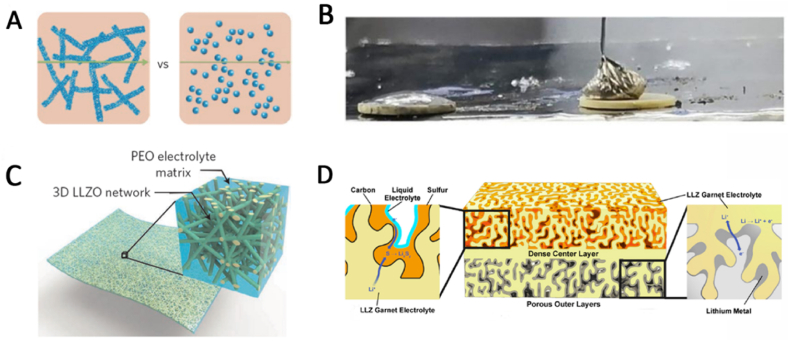
Polyacrylonitrile solid electrolyte blended with LLTO nanowires offers a continuous conduction pathway(A) [151]. Images of the melted Li metal on top of the LLZT surface after rub coating and stacking(B) [152]. Schematic showing the LLZO-nanowire-blended PEO solid electrolyte(C) [153]. Schematic showing the full cell with porous and dense electrolyte (D) [154].

In order to achieve these goals, solid-state polymer and inorganic ceramic composite electrolytes have been developed, which can overcome the shortcomings of ceramics and polymers, combine the characteristics of flexibility and inhibition of lithium dendrite growth, and can be applied in all-solid-state batteries. Recently, Zhou et al. invented a solid electrolyte with a polymer-ceramic-polymer sandwich structure, in which Li_1.6_Al_0.5_Ti_0.95_Ta_0.5_(PO_4_)_3_(LATP) and cross-linked poly (ethylene glycol) methyl ether acrylate are mixed together to form a soft sheet, This sandwich-type composite electrolyte can effectively solve the problem of high impedance at the interface between electrode and ceramic by adhesion of lamellae and bulk LATP ceramics, and can use the mechanical strength of ceramics to inhibit the growth of lithium dendrites [150]. In addition, one-dimensional Li_3x_La_2/3–x_TiO_3_(LLTO) nanofibers mixed with polyacrylonitrile (Fig. 8(A)) can provide continuous lithium ion conduction and greatly improve the room temperature lithium ion conductivity of the electrolyte (about 0.1 mS cm^−1^) [151]. Duan et al. use fresh lithium metal without without Li_2_CO_3_ to reduce the impedance at the interface between lithium metal and ceramic (Fig. 8(B)) [152]. Similarly, a composite electrolyte formed by mixing nano-garnet Li_7_La_3_Zr_2_O_12_ (LLZO) solid electrolyte with polyvinyl oxide (PEO) can achieve similar results (Fig. 8(C)) [153]. Hitz and his co-wokers use the dense and porous LLZO combination to make the big contact area between electrode and ceramics which enhance the electrochemical performance (Fig. 8(D)) [154]. Nonetheless, the high density and bad processability restrain the further development of solid electrolyte.

## 5 Perspective

**Fig. 9:**
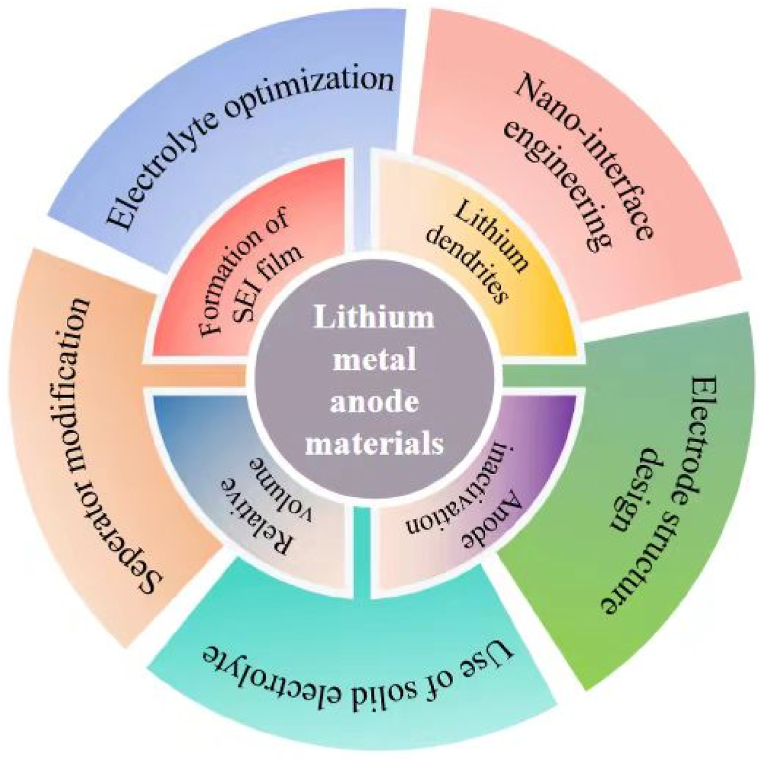
The summarize of lithium metal anode and its challenge for large-scale application.

In recent years, lithium metal anode has attracted the researchers’ attentions due to its high specific capacity and low potential which can make the lithium battery reach the energy density limit of the battery system. But the problems such as active solid electrolyte interface (SEI) film consume and lithium dendrites and so on make the lithium metal anode face challenges to develop. Therefore, researchers take electrolyte optimization, nano-interface engineering, electrode structure design, separator modification and use of solid electrolyte to overcome these problems (Fig. 9). In my opinion, separator modification is the most feasible way for practical application in the future. The coating way of separator modification is a common way in the commercial battery. Boehmite and Al_2_O_3_ are largely used as coating agent so that the lithium dendrites suppressor material can also be coated on the separator with the current technology.

Although, these methods promote the lithium metal anode to obtain great development in science area. there are still a lot of engineering issues in the commercial use. The large scale of lithium metal also face the engineering problems such as the mechanical stress by coiling and slitting, electrolyte consume and volume swelling stress. on the other hand, the manufacturing equipment and environment of lithium metal anode can't be suitable with that of lithium-ion battery. Although SES produced 100 Ah lithium metal cell A in 2021, the cell B with the more capacity can't be report until now. Therefore, the commercial use of lithium metal battery has a long way to run. The battery consistency and reproducibility of manufacturing is the key point and requires abundant supply chain. We believe that the lithium metal battery will be put in practice with the more effort by industrial capital.

The lithium metal anode is the key point of next generation battery with the highest energy density. As a result, the 400 Wh kg^−1^ lithium battery with lithium metal anode can't be formed large-scale applications. The scientists and engineers need to solve practical issues in next 10 years.

## Ethical approval

Not applicable.

## Funding

This work was supported by the 10.13039/501100003787Natural Science Foundation of Hebei Province (no. E2020209183), basic expenses for scientific research in North China University of Science and Technology (No. JQN2023024),the project of High level group for research and innovation of School of Public Health, North China University of Science and Technology (KYTD202309).

## Availability of data and materials

All relevant data are within the manuscript and its Additional files.

## CRediT authorship contribution statement

**Ran Tian:** Writing – review & editing, Writing – original draft, Supervision, Data curation, Conceptualization. **Jingyu Jia:** Writing – original draft. **Meixiang Zhai:** Writing – review & editing. **Ying Wei:** Writing – review & editing. **Xinru Feng:** Writing – review & editing. **Ruoqi Li:** Writing – review & editing. **Jinyan Zhang:** Writing – review & editing. **Yun Gao:** Writing – review & editing.

## Declaration of competing interest

The authors declare the following financial interests/personal relationships which may be considered as potential competing interests:

Tian Ran reports financial support was provided by North China University of Science and Technology. If there are other authors, they declare that they have no known competing financial interests or personal relationships that could have appeared to influence the work reported in this paper.
